# Supplementary material for: Long-Term High-Fat Diet Limits the Protective Effect of Spontaneous Physical Activity on Mammary Carcinogenesis
Source: Int J Mol Sci. 2024 Jun 5;25(11):6221. doi: 10.3390/ijms25116221 (PMC11172547; doi:10.3390/ijms25116221)
Supplement: Supplementary file 1 [file ijms-25-06221-s001.zip › ijms-2991835-supplementary.pdf]

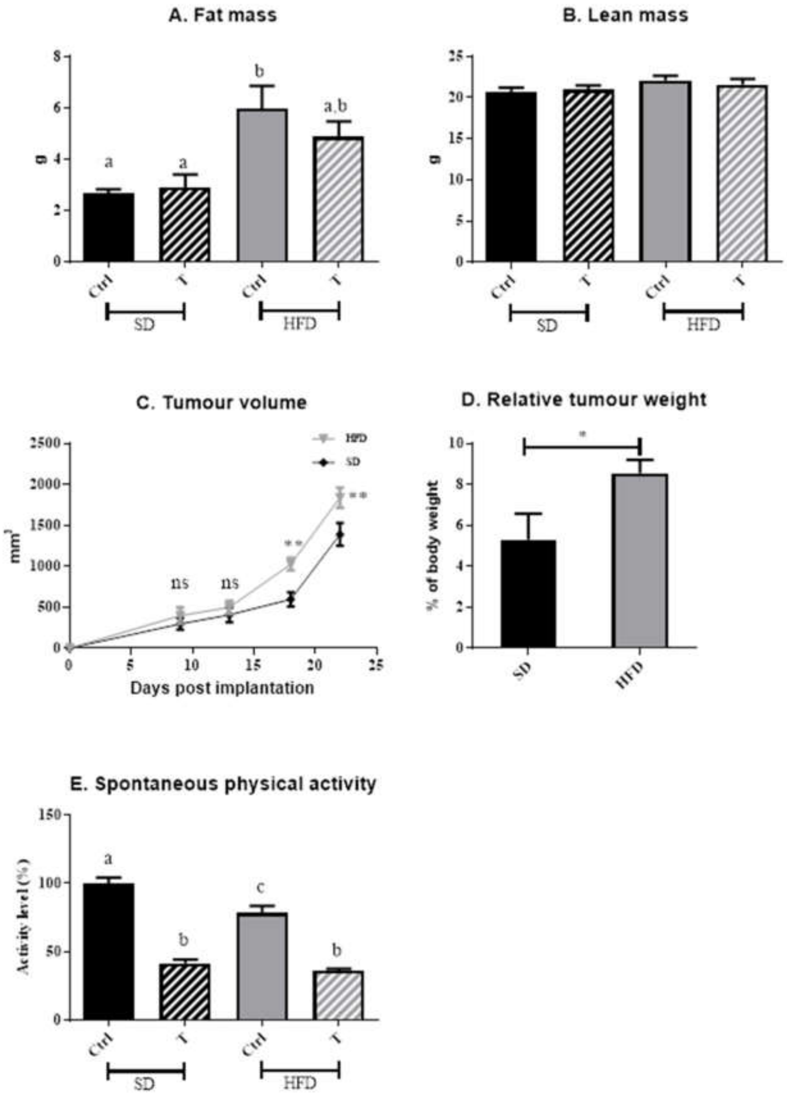

Figure S1. Difference in body composition and tumour growth between mice under standard diet and high-fat diet. This experimentation was performed on aged ovariectomized C57BL/6 fed with a short-term (12 weeks) standard control or the same high-fat diet used in the present study. Results are mean ± SEM (n=10/group). (**A, B and E**) Data were analyzed by two-way ANOVA repeated measures followed by a Bonferroni multiple comparison test. Level of significance was set at 0.05. Different letters shown representative data: a ≠ b ≠ c, $p < 0.05$. (**C**) Data were analyzed by ANOVA repeated measures. Representative data are shown: * $p < 0.05$, ** $p < 0.01$, SD vs. HFD. (**D**) Data were analysed by a Mann and Whitney test. Representative data are shown: * $p < 0.05$. Mahbouli, Sinda, Adrien Rossary, Rachida Nachat-Kappes, Kristell Combe, Samar Basu, Marie-Paule Vasson. Leptin induces in vitro and in vivo a lipid peroxidation and an inflammatory response, in neoplasic mammary epithelial cells. » Oral communication presented at the 11th Biennial ISSFAL Congress, Stockholm, Sweden, 28 juin 2014.
